# The Impact of Oral Health on Respiratory Viral Infection

**DOI:** 10.3390/dj9040043

**Published:** 2021-04-13

**Authors:** Akio Tada, Hidenobu Senpuku

**Affiliations:** 1 Department of Health Science, Hyogo University, Kakogawa 675-0195, Japan; 2 Department of Bacteriology, National Institute of Infectious Diseases, Tokyo 162-8640, Japan; hsenpuku@nih.go.jp

**Keywords:** SARS-CoV-2, influenza virus, oral cavity, saliva

## Abstract

Influenza virus and severe acute respiratory syndrome coronavirus (SARS-CoV-2) have caused respiratory diseases worldwide. Coronavirus disease 2019 (COVID-19) is now a global health concern requiring emergent measures. These viruses enter the human body through the oral cavity and infect respiratory cells. Since the oral cavity has a complex microbiota, influence of oral bacteria on respiratory virus infection is considered. Saliva has immune molecules which work as the front line in the biophylactic mechanism and has considerable influence on the incidence and progression of respiratory viral infection. Salivary scavenger molecules, such as gp340 and sialic acid, have been reported to exert anti-influenza virus activity. Salivary secretory immunoglobulin A (SIgA) has potential to acquire immunity against these viruses. Biological features of the oral cavity are thought to affect viral infection in respiratory organs in various ways. In this review, we reviewed the literature addressing the impact of oral conditions on respiratory infectious diseases caused by viruses.

## 1. Introduction

Global pandemics of respiratory infectious diseases such as coronavirus disease-2019 (COVID-19) and bird flu have been enormous health crises to people over the worldwide. Since the oral cavity is in the vicinity of the respiratory tract, where these viruses enter and replicate, oral health status is thought to impact the initiation, progression, and pathology of respiratory infectious diseases. The oral microbiota is thought to be one of the factors that influence respiratory virus infection. Coinfection with influenza virus and bacteria has been noted because it may cause severe morbidity and mortality [1]. Regarding severe acute respiratory syndrome coronavirus 2 (SARS-CoV2), the impact of oral bacterial infection on COVID-19 has been discussed [2,3].

On the other hand, immune function of the oral cavity has been known to affect oral infection. Saliva is a key component of the host defense against infection in the mouth and full of immune materials. Salivary scavenger and agglutinin are responsible for innate immunity in the oral cavity [4]. Secretory immunoglobulin A (SIgA), which plays a critical role in mucosal immunity, is secreted from the salivary gland [5].

Evidence addressing the associations between oral health and the prevention of incidence and aggravation of respiratory infections has not been sufficiently reviewed. The elucidation of the influence of oral health on respiratory viral infection diseases will illustrate the way that dental health care can contribute to the prevention of incidence and progression of these diseases.

The aim of the present study was to collect and review findings addressing the impact of oral condition and oral care on SARS CoV-2 infection and influenza viral infection and to obtain fundamental knowledge to provide effective oral health care and treatment to patients with these respiratory diseases.

## 2. Methods

Literature search was based on databases PubMed. Date of last search is 21st March 2021. Authors used following combinations of terms: [“influenza virus”, and “oral bacteria”], [“influenza virus”, “saliva”, and “SIgA”], [“influenza virus”, “saliva”, and “sialic acid”], [“SARS-CoV2”, “saliva”, and “SIgA”]. Authors also hand-searched for relevant papers and reviews to identify journal articles that might not have been captured through our search strategy. Only articles in English were included. The authors selected eligible literature by consent. With regard to [“SARS-CoV2”, “saliva”, and “SIgA”], studies concerning diagnosis using saliva sample were excluded.

## 3. The Influence of Oral Health on Influenza Virus Infection

### 3.1. Direct Influence of Oral Bacteria

#### 3.1.1. Apoptosis Induced by *Porphyromonas gingivalis*

The apoptosis induced by viral infection is generally recognized to have a role as a defense mechanism that prevents viral proliferation by programmed cell death. Infection with influenza virus causes significant cell death within the upper and lower respiratory tract and lung parenchyma. Most apoptotic induction depends on an intracellular cascade consisting of catalytic activation of cysteine-dependent aspartate-directed proteases (caspases). Chen et al. reported that the combination of *P. gingivalis* and H1N1 infection in lung epithelial cells may promote the production of inflammatory cytokines and increase NO production, leading to increased levels of apoptosis in lung epithelial cells via the Bcl-2/Bax/caspase-3 signaling pathway [6]. It is considered that co-infection with *P. gingivalis* and influenza virus highlighted the production of inflammatory cytokines and NO through Bcl-2/Bax/caspase-3 signaling, consequently increasing apoptosis levels. On the other hand, infection with both *P. gingivalis* and influenza A virus (IAV) temporarily inhibited apoptosis in respiratory epithelial cells, which may be related to the initiation of autophagy [7]. The regulation of the apoptosis by influenza virus and *P. gingivalis* may be complex and depend on the stage of viral infection.

Enhancements of respiratory viruses by *P. gingivalis* are in vitro phenomena, and there are some barriers to be observed in vivo. Oral bacteria, including periodontopathogens, cannot move to respiratory organs easily in individuals with normal swallowing function. Nishioka et al. reported that oral streptococci were isolated from the bronchoalveolar lavage fluid (BALF) of acute respiratory distress syndrome (ARDS) patients, suggesting the transfer of oral bacteria from the oral cavity to the lower respiratory tract [8]. Swallowing function may have influenced the distribution of oral bacteria in those patients.

#### 3.1.2. Increase of Influenza Virus Proliferation Induced by Oral Bacteria

Some studies have demonstrated that oral streptococcal species increase influenza virus proliferation under in vitro conditions [8,9]. However, similar phenomena in the oral cavity require a large amount of such bacterial species or long-term stay of virus in the oral cavity.

### 3.2. Depression of Immunity Induced by Periodontal Disease

Given that periodontal pathogens influence influenza virus infection, the most plausible explanation is that local inflammatory reactions in patients with severe periodontal disease spread systemically and decrease immunity. *Aggregatibacter actinomycetemcomitans* produces a factor that downregulates T-cell proliferation and cytokine production at local defense sites [10,11]. Anaerobic gram negative bacteria including *P. gingivalis* greatly inhibit T- and B-cell proliferation, inducing immunosuppression [12]. Oral mucosal epithelial cells and oral fibroblasts that were impaired by periodontopathogens secrete butyric acid, which induces inflammatory reactions and the apoptosis of immunocompetent cells in local tissues. Lowering the immune response in the gingiva is assumed to induce active inflammatory cytokine production and increases inflammatory mediators (CRP, IL-6, TNF-α) in the blood, which consequently, results in a decrease in the immunological defense system. A decrease in immunity induced by periodontitis pathogens is thought to make humans vulnerable to influenza virus infection.

### 3.3. Inhibition of Influenza Virus Proliferation by Salivary Immunity

#### 3.3.1. Innate Immunity

Saliva plays a key role in protecting the host from a wide variety of pathogen infections including viruses and bacteria in the oral cavity. Many biomolecules in saliva have antiviral activities against specific viruses [13]. Saliva can control virus infection through many different biomolecules, including mucins, antibodies, and antiviral proteins. These biomolecules are supplied in a continuous flow of fluid. Whole saliva or parotid or submandibular/sublingual secretions from healthy donors inhibited IAV based on haemagglutination inhibition and neutralization assays [14]. The extent of inhibition of influenza virus infection by saliva depends on virus species [15]. Among purified salivary proteins, MUC5B, scavenger receptor cysteine-rich glycoprotein 340 (salivary gp-340), histatins, and human neutrophil defensins (HNPs) inhibited IAV at the concentrations present in whole saliva [16]. The antiviral activity of GP340 is significant against IAV and human immunodeficiency virus (HIV1), by contrast, GP340 has little or no anti-viral activity against herpes simplex virus (HSV), HIV-2, or simian immunodeficiency virus (SIV) [16].

Human saliva contains the sialic acid type corresponding to the binding preference of seasonal influenza viruses [17]. Elderly individuals with T2DM and liver disease had significantly lower levels of the expression of the terminal α2-3-linked sialic acids [18]. This finding may partly explain that having chronic disease are associated with serious influenza-related complications, including elevated mortality. Gilbertson et al. showed that anti-influenza activity of infant saliva is associated with sialic acid-containing molecules in infants aged 1–12 month [19]. Salivary sialic acid is thought to work as an innate immunization protein to protect infants from influenza virus infection, who are not immunized against influenza virus.

#### 3.3.2. Humoral Immunity

**Figure 1:**
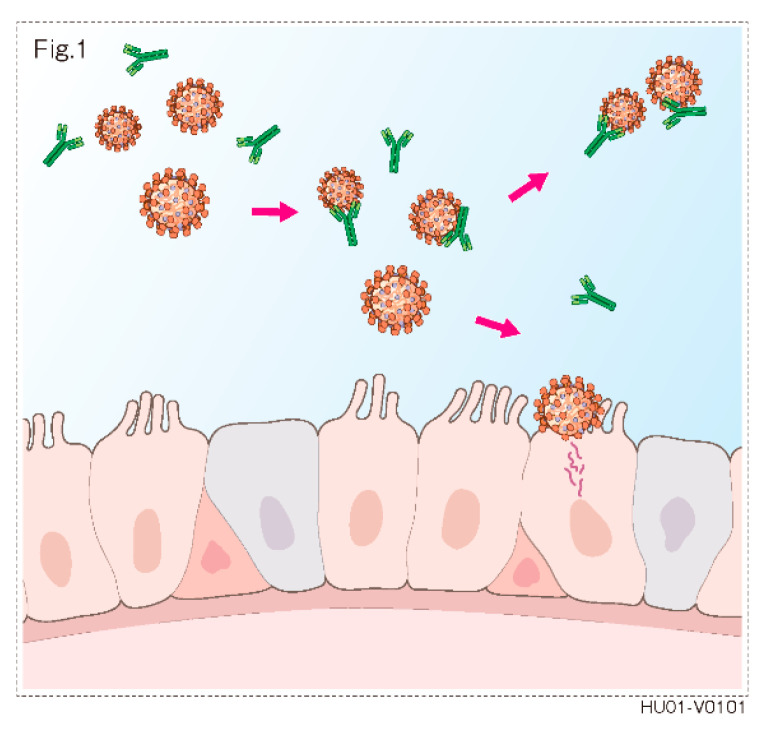
SIgA in saliva prevents the invasion of pathogens such as bacteria and viruses by which SIgA inhibits pathogen attachment and settlement to the mucous membrane by binding and aggregating pathogens.

SIgA is a subclass of Immunoglobulin A (IgA), an antibody that plays a critical role in mucosal immunity. SIgA is the main immunoglobulin found in mucous secretions from salivary glands. SIgA in saliva works as an initial defense that prevents the invasion of pathogens such as bacteria and viruses by which SIgA inhibits pathogen attachment and settlement to the mucous membrane by binding and aggregating pathogens (Figure 1).

SIgA antibodies have neutralization potential pathogens at the entrance site before they can attach to epithelial cells and overcome the epithelial surface. Considerable levels of Influenza virus specific SIgA was secreted in saliva, suggesting that saliva works as humoral immunity against influenza virus [20,21]. The development of mucosal vaccines that aim to induce influenza virus-specific IgA has been working on. Langley reported that nasally administered inactivated trivalent influenza vaccine significantly increased salivary secretory IgA in healthy adults aged 18–64 years [22]. Practical realization of mucosal vaccine for influenza virus will enhance the importance of immunity in the oral cavity.

### 3.4. Epidemiological Study

No cross-sectional study investigating the association between a history of influenza and periodontal disease among community dwelling people or case–control study comparing periodontal health between patients with influenza and people in good respiratory health has been published. In a randomized controlled trial (RCT) study of day care service users, the experimental group with an intervention of professional oral health care had a significantly lower prevalence rate of influenza and lower levels of neuraminidase and trypsin-like protease in saliva than the control group [23].

No other study has examined the association between influenza virus infection and oral care. The impact of oral health on influenza virus infection reviewed here suggests the necessity of investigating the association between the severity of periodontitis and influenza virus infection and between saliva secretion and influenza virus infection.

## 4. COVID-19 and Oral Health

### 4.1. Saliva and SARS-CoV-2

SARS-CoV-2 binds the receptor, angiotensin-converting enzyme 2 (ACE2) on the surface of multiple cell types [24,25,26]. Salivary gland cells are found to have ACE2 and be infected by SARS-CoV-2 [27,28]. Other oral tissue cells such as osteoblast and osteoclast of alveolar bone, fibroblast, gingiva, and the periodontal ligament [29,30]. ACE2 expression in the nasal epithelium was lower in children than in adults and was considered to increase with age [31]. This ageing alteration is presumed to apply to the salivary gland. The lower expression of ACE2 may be responsible for the lower COVID19 incidence rate in children.

#### 4.1.1. Anti-Viral Activity of Saliva

Saliva has liquidity and exerts a function of washing materials, including virus, away. People with a small amount of saliva have a risk of insufficient ability to wash away virus. Although saliva containing high virus load could be an infection source, increased secretion of saliva is expected to dilute virus in saliva and decrease the risk of virus transmission.

As mentioned above in this review, salivary components include anti-viral molecules such as cathelcidin, lactoferrin, lysozyme, mucin, peroxidase, salivary agglutinin (gp340, DMBT1), SLPI, and α and β defensins have been reported as salivary antiviral components [32]. Salivary anti-viral components inhibit the growth of various viruses in the oral cavity, such as HSV, HIV, vesicular stomatitis virus (VSV), Epstein-Barr virus (EBV), human papilloma virus (HPV), Ebola virus, human herpes virus (HHV), measles morbillivirus, adenovirus, rabies virus, hepatitis A virus (HAV), hepatitis C virus (HCV), influenza virus, and Hantavirus [16,32,33,34,35]. Furthermore, virus-specific SIgA is induced in saliva after virus infection and produces anti-viral effects.

#### 4.1.2. Possibility of the Inhibition of SARS-CoV2 by Saliva

Although the anti-SARS-CoV-2 effect of saliva has not yet been reported, it is quite conceivable that salivary immunity works to inhibit infection of SARS-CoV-2. Since SARS-CoV-2 infect salivary gland cells, virus-specific SIgA must be secreted in saliva. In COVID-19 patients, a correlation (r = 0.4405) between salivary IgA levels and COVID-19 disease severity was found [36]. Sterlin et al. reported that most of saliva samples from SARS-CoV2 infected patients neutralized SARS-CoV2 pseudotyped viral particles with a significant correlation between neutralization activity and anti-RBD IgA titers (*r* = −0.796, *p* < 0.008) [37]. SARS-CoV2 specific IgA monoclonal antibody exhibited strong neutralizing activity against SARS-CoV2, suggesting the important role of IgA [38]. These evidence elicit the potential of SIgA in saliva to prevent SARS-CoV2 infection. In a murine model, the production of SARS-CoV-specific serum IgG and SIgA was detected in saliva following intranasal immunization after SARS-CoV infection [39]. Recently available mRNA vaccines presented that antibody to S protein, and the Receptor Binding Domain of SARS-CoV-2 were detected in saliva [40]. It is expected that IgA antibody is produced in salivary glands, and have an important role in suppression of SARS-CoV-2 proliferation in salivary glands and in preventing the excretion of the virus into saliva.

SIgA secretion is known to decrease with age. Middle-aged and older adults had lower saliva secretion than younger adults [41]. Lower salivary SIgA was significantly related to increasing age [42,43]. A decrease in SIgA secretion with ageing is thought to permit submucosal pathogen entry, consequently causing upper respiratory disease. The difference in vulnerability to Sars-CoV-2 infection between younger people and elderly people may depend on antiviral activity in the salivary gland and saliva of individuals. A study reported a significant positive correlation between age and peak viral load [44], which may result from decreased antiviral activity of saliva with age.

Since saliva is a source of SARS-CoV-2 infection, sufficient attention should be paid to droplet infection through saliva. Nevertheless, an increase in saliva secretion and SIgA concentration in saliva is thought to decrease SARS-CoV-2 infection. Chewing has been reported to have a positive impact on saliva secretion [45,46,47,48]. The number of chewing cycles is effective in increasing saliva flow. A few studies addressing the association between exercise and salivary SIgA have been reported. Twelve months of exercise training significantly increased the level of salivary SIgA among middle-aged adults [49]. Elderly people who walked 7000 steps/day had higher SIgA levels than those who walked 3000 steps/day [50]. Staying home for fear of infection may result in a decrease in SIgA.

Few studies have investigated the association between saliva secretion and saliva SIgA and SARS CoV-2 infection. Further evidence addressing the influence of saliva on SARS CoV-2 infection is required.

Bioinfomatic studies suggested that the SARS-CoV-2 spike protein is likely to bind sialic acid glycans [51,52]. A domain in the cap or knob of the SARS-CoV-2 spike is involved in the non-covalent binding of host sialic acid glycans. SARS-CoV-2 is expected to use sialic acid as a receptor in addition to ACE2. Salivary sialic acid may inhibit SARS-CoV-2 infection, like influenza virus.

#### 4.1.3. The Use of Saliva as a Possible Way of COVID-19 Diagnosis

SARS-CoV-2 exhibits high infectivity from human to human. More correct and safe diagnosis for SARS-CoV-2 infection is necessary to avoid virus transmission to healthy individuals and health care providers. Sensitivity and specificity for SARS-CoV-2 detection of saliva specimen was comparable to that of nasopharyngeal and throat swabs [53,54]. The use of saliva has some advantage of being rapid, less invasive, and decreasing the possibility of healthcare personnel to SARS-CoV-2, it has a potential to become an important tool for diagnosis of SARS-CoV-2 infection.

### 4.2. Association between COVID-19 and Periodontitis

A case–control study demonstrated an association between periodontitis and the severity of COVID-19 infection [55]. It has been found that there is a clear correlation between these two diseases and this correlation is dual-direction: The exacerbation of COVID-19 occurred by an influence of periodontitis and the cytokine storm syndrome caused by the virus could accentuate periodontitis. During SARS-CoV-2 infection, ACE-2 is under expressed and cannot form the ACE2–angiotensin1–7–Mas receptor axis, resulting in an increase in inflammatory cytokines such as interleukin-6, interleukin-7, tumor necrosis factor alpha, interleukin-2, interleukin-1 beta, monocyte chemoattractant protein-1, and transforming growth factor-beta, associated with a periodontal disease [56]. The changes in the expression of cytokines are thought to explain part of the association between periodontitis and systemic chronic diseases [57,58,59,60,61,62,63,64,65,66,67], which suggests a wide and profound impact of periodontal disease on systemic health.

## 5. Conclusions

In this review, we discuss the possible influence of oral health status on respiratory viral infection from various points of view. Immunity in saliva is, in particular, thought to have considerable impacts on the incidence and progression of respiratory viral infection. Parts of antiviral mechanisms against influenza virus and SARS-CoV-2 by immunity in saliva are similar. Little is known about the mechanisms by which various factors inhibit or exacerbate viral infection in the oral cavity. It is important for prevention of viral infection by oral care based on evidence to draw perspective of the role of the oral cavity in the virus infection. This review shows a proper direction toward this goal.

## Data Availability

Not applicable.
